# Questionnaire on Perception of Threat from COVID-19

**DOI:** 10.3390/jcm9041196

**Published:** 2020-04-22

**Authors:** María del Carmen Pérez-Fuentes, María del Mar Molero Jurado, Nieves Fátima Oropesa Ruiz, África Martos Martínez, María del Mar Simón Márquez, Iván Herrera-Peco, José Jesús Gázquez Linares

**Affiliations:** 1 Department of Psychology, Faculty of Psychology, University of Almería, 04120 Almería, Spain; foropesa@ual.es (N.F.O.R.); amm521@ual.es (Á.M.M.); msm112@ual.es (M.d.M.S.M.); 2 Department of Psychology, Faculty of Psychology, Universidad Politécnica y Artística del Paraguay, Asunción 1628, Paraguay; 3 Health Sciences Collegue, Alfonso X El Sabio University, 28691 Madrid, Spain; iherrpec@uax.es; 4 Department of Psychology, Universidad Autónoma de Chile, Providencia 7500000, Chile

**Keywords:** adults, COVID-19, perceived threat of disease, psychometric properties

## Abstract

The ravages caused by the disease known as COVID-19 has led to a worldwide healthcare and social emergency requiring an effective combined effort from everyone to reduce contagion. Under these circumstances, the perception of the disease is going to have a relevant role in the individual’s psychological adjustment. However, at the present time there is no validated instrument for evaluating adult perception of threat from COVID-19. Considering the importance of perception or representation of the disease in a state of social alert, our study intended to validate an instrument measuring the psychological process of the disease caused by the coronavirus (COVID-19). In view of the above, this study evaluated the factor structure and reliability of the version of the Illness Perception Questionnaire (IPQ) for COVID-19 in a sample of adults. The sample consisted of 1014 Spanish adults (67.2% women and 32.8% men). The exploratory and confirmatory factor analyses supported a unidimensional model of the scale, which was the one that showed the best fit and explained 43.87% of the variance. This brief version has adequate psychometric properties and may be used to evaluate the perception of threat from COVID-19 in an adult Spanish population. The validation of this instrument contributes to progress in representation of COVID-19 in our culture.

## 1. Introduction

The current outbreak of COVID-19 caused by a new coronavirus known as Severe Acute Respiratory Syndrome Coronavirus 2 (SARS-CoV-2) [1] was located for the first time in Wuhan (China) in December 2019. The symptoms associated with COVID-19 include fever, cough, shortness of breath, diarrhea, and fatigue. Complications include pneumonia, acute severe respiratory distress syndrome, renal insufficiency, or even death in certain cases [1,2]. 

The rapid spread of the disease, which was already observed during the months of December 2019 and January 2020, led the World Health Organization to define COVID-19 as a global public health emergency on January 30, 2020 [3]. There is a strong probability that the coronavirus that causes this disease, known as SARS-CoV-2, has a zoonotic origin. If this hypothesis is confirmed, veterinarian coronavirologists could be a reference for treatment of infections by SARS-CoV-2 in humans [4]. At the time of writing, March 2020, the high number of cases and the many countries affected define COVID-19 as a global pandemic such that on April 19, 2020 [5] figures related to COVID-19 had surpassed 2,281,714 confirmed cases and over 159,511 deaths associated with COVID-19 on five continents. These data show how highly infectious the SARS-CoV-2 virus, which causes COVID-19, is around the world. The shortage of resources that turns this situation into a worldwide healthcare and social emergency demands the effective combined effort from everyone and of all the organizations involved [6,7]. Neither should it be forgotten that the search for an effective treatment, which is not yet available, requires coordination without precedent of healthcare professionals and the scientific community [8].

Although many clinical studies are underway, those analyzing the impact on psychological well-being of the population are not as numerous [9]. In previous pandemics, studies showed that healthcare professionals were under strong stress from fear of becoming ill, spreading the disease to their families, and the heavy work load [10,11]. Several authors have found that perceived threat of the disease can cause severe psychological maladjustment, such as depression, anxiety and stress [9,11,12], which are involved in the emotional exhaustion of healthcare employees [13,14]. Keeping in mind the importance of perception or representation of the disease in situations of social alert, our study attempted to validate an instrument for measuring the psychological processing of the disease caused by the coronavirus (COVID-19). 

### Perceived Threat from COVID-19 

The disease perception model focuses on the perceptions, constructions, or representations one has about experience with a disease, its identity, consequences, treatment, causes, duration, and cure [15,16,17]. This conception of a disease influences one’s interpretation of the symptoms and is conditioned by experience with previous pathologies, as well as by the social and cultural context [18,19,20]. Quiceno and Vinaccia [21] showed that representation of a disease influences prevention behavior, reactions to the symptoms, adherence to treatment when diagnosed, and future expectations for health. 

Therefore, one’s perception of a disease depends on interpretation of experience, transfer of this interpretation to active behavior, response to social reactions, and personal meaning attributed to the experience. In the situation of imminent alarm in which global society is now immersed with the arrival of COVID-19 and its effect on health, adult perception of the disease acquires considerable significance, as the measures taken by governments involve changes in habits and lifestyles. 

The instruments employed in evaluating the perception of disease as a threat may be differentiated by their theoretical foundations [15,19,20]. The scale most widely used is the Illness Perception Questionnaire (IPQ), validated with a sample of patients with chronic diseases (asthma, liver, and diabetes), is made up of 38 items. The nine-item brief version (BIP-Q) [16], has also been widely used [12,22,23]. The original questionnaire measures identity, cause, timeline, consequences, and control/cure. Studies have proven the usefulness of both the original version and the brief questionnaire for exploring perception of the disease in different areas of health [12,16,23,24]. Later, Moss-Morris et al. [17] revised the original questionnaire to extend it to the cognitive and affective dimensions of disease perception in the Illness Perception Questionnaire Revised (IPQ-R) with 70 items. In this version, the “timeline” subscale enables differentiation between acute/chronic and episodic and the “control/cure” subscale between personal control/cure and what can be attributable to the treatment. The authors further added two new subscales which evaluate “emotional representation” on one hand and “coherence” on the other.

Leventhal et al. [25,26] based illness perception on the Common Sense Self-Regulation Model (CSM), which emphasizes empowering the individual with behavior control. From this approach, the perception of illness involves several different processes which explain: 1) how people perceive a threat to their health; 2) how they generate a mental representation and associated emotions with that threat; and 3) how they start up different plans of action for their regulation and coping, which they constantly revised based on feedback received on their efficacy and the progression of the threat. 

In view of the relevance of measuring and evaluating the perception of threat from COVID-19 in the population, this study validated the Brief Illness Perception Questionnaire version BIP-Q5 [20] employed, in this case, to evaluate perceived threat from COVID-19.

## 2. Method

### 2.1. Participants

The sample was made up of 1043 Spanish adults from 19 autonomous regions. The questionnaire had control questions for detecting random or incongruent answers leading to the elimination of 29 subjects, so that the final sample was comprised of 1014 persons of whom 67.2% (*n* = 681) were women and 32.8% (*n* = 333) men, with a mean age of 39.88 (standard deviation (SD) = 12.35) and 42.92 years (SD = 12.33), respectively. The mean sample age was 40.87 (SD = 12.42) ranging from 18 to 76.

### 2.2. Instruments

The Brief Illness Perception Questionnaire, version BIP-Q5 [16], made up of nine items and translated into Spanish by Pacheco-Huergo et al. [27], was used in this study. In this shorter version of the BIP-Q, Items 3, 4, and 7 were eliminated, and Item 9 was an open-ended question. The BIP-Q5 therefore consists of five items on perception of threat from illness, where participants rate their agreement with the statements on a Likert-type scale from 0 to 10. The test provides an overall score on the representation of the illness. The higher the score is, the greater the perception of the illness as a threat. This brief version of the questionnaire has adequate psychometric properties [24]. The BIP-Q has also shown acceptable reliability indices with large-scale adult populations in several different countries [16,23,28,29]. Other versions of the questionnaire have robust validations with Spanish samples [15,30]. In this study, the internal consistency coefficient was acceptable with a Cronbach alpha of 0.663. This study is a pioneer in exploration of the psychometric properties of the instrument for COVID-19 in a general adult Spanish population. The BIP-Q5 was therefore adapted to this disease (for example, “How much are you worried about being infected by the coronavirus (COVID-19)?” or “How much does infection by the coronavirus (COVID-19) affect you emotionally?” (That is, does it make you feel furious, afraid, angry or depressed?)”.

### 2.3. Procedure

This cross-sectional study was carried out in a sample found by snowball sampling, which was publicized on social networks and by texting during the first week of confinement of the Spanish population from March 18 to 23, 2020. The participants filled out the tests individually in an estimated mean time of 5 to 10 minutes. In all cases, ethical research standards were complied with by providing information on the project and requesting consent to participate. The study was approved by the University of Almeria Bioethics Committee (Favorably reported on March 24, 2020).

### 2.4. Data Analysis

Data analyses were performed in two stages following the validation steps by Pérez-Fuentes et al. [31]. In the first stage, the BIP-Q5 structure was studied. For this purpose, the sample was divided at random into two independent homogeneous subsamples. The first (*n* = 505) was used as the calibration sample for confirmatory factor analyses (CFA) of the original Threat Perception model. The confirmatory factor analysis for the original model used the following fit indices as measures: χ2/df, comparative fit index (CFI), Tucker-Lewis index (TLI), and root mean square error of approximation (RMSEA) with a confidence interval (CI) of 90%. For the χ2/df, values below five were considered acceptable [32]; for the CFI and incremental fit index (IFI), a value over or near 0.90 were acceptable; and for the RMSEA values below or very near 0.08 were considered acceptable [33]. As a general rule, good fit of the model would be when: 2/DF ≤ 3; TLI > 0.90; CFI > 0.95; and RMSEA ≤ 0.05. The appropriate respecifications were made to the model proposed, which had shown good fit indices, considering theoretical and statistical criteria (change indices, errors of estimation, standard errors of measurement), but it did not improve the original model. The Akaike information criterion [34] was used for model selection. The second subsample (*n* = 508) was used as the validation sample for the respecified model. The Cronbach’s alpha [35], Spearman–Brown formula, and intraclass correlation coefficient were used for the reliability analysis of the new scale.

Finally, in the second stage, an analysis was performed to find out whether the factor structure was invariant across sex (male/female). In the first place, goodness of fit of these structures was tested separately in both subsamples (Models M0a-Male and Model M0b-Female). The resulting four nested models were evaluated: (a) Model 1, both subsamples were considered together with free estimation of the parameters; (b) Model 2, metric invariance is shown; (c) Model 3, shows scalar invariance; and (d) Model 4, strict invariance. There was no criterion of consensus for determining the criteria to be used to evaluate the difference in fit between the different nested models [36]. The ΔCFI was used for evaluating fit. The model was interpreted as completely invariant if the ΔCFI was below 0.01 [37]. 

The analyses were performed with the SPSS statistical package version 23.0 for Windows and the AMOS 22 program.

## 3. Results

### 3.1. Preliminary Analyses

In the first place, the data showed that the distribution of the items on the BIP-Q5 were within the limits of normality according to the Finney y DiStefano [38] criterion, in which 2 and 7 are the maximum permissible values for skewness and kurtosis, with maximums in our case of 2.1 and 3.8, respectively. In the exploratory factor analysis, principal components extraction was used with direct Oblimin rotation (Kaiser-Meyer-Olkin; KMO = 0.71) which enabled correlation between factors. Table 1 shows descriptive statistics of the calibration sample (*n* = 505).

### 3.2. Exploratory Factor Analysis of the Original BIP-Q5 Model

**Figure 1:**
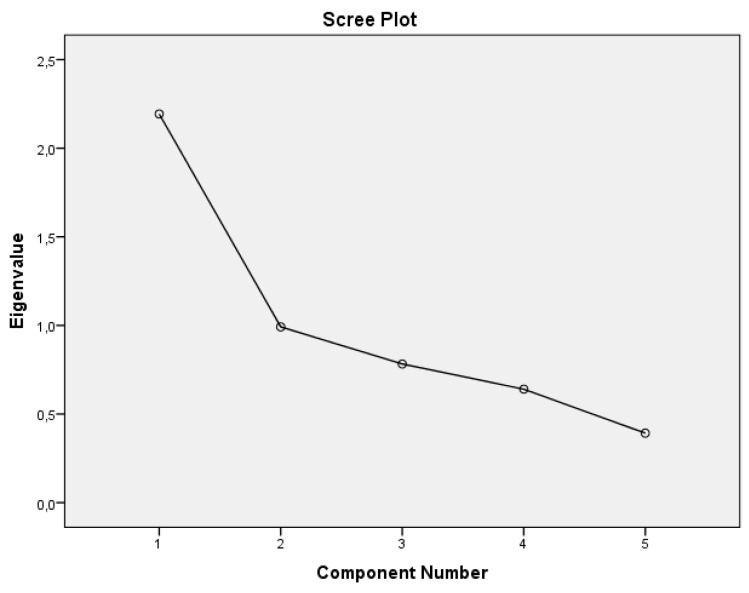
Scree plot of factor analysis of the original Brief Illness Perception Questionnaire (BIP-Q5) model.

The principal components analysis revealed the existence of one component with eigenvalues over 1. The scree plot showed no need for rotation with only one factor (Figure 1). Thus, in Table 2 only one component is presented, and that factor (perception of threat from COVID-19) was comprised of five items, all of them with weights over 0.60, except for Item 3 “How much do you feel symptoms of infection by coronavirus?”, and these explain 43.87% of the variance.

### 3.3. Confirmatory Factor Analysis of the BIP-Q5 Model for COVID-19

Fit of the questionnaire models is presented in Table 3 according to the original BIP-Q5 model (analyzing the one-factor and two-factor models of the BIP-Q5), adapted to COVID-19. 

**Figure 2:**
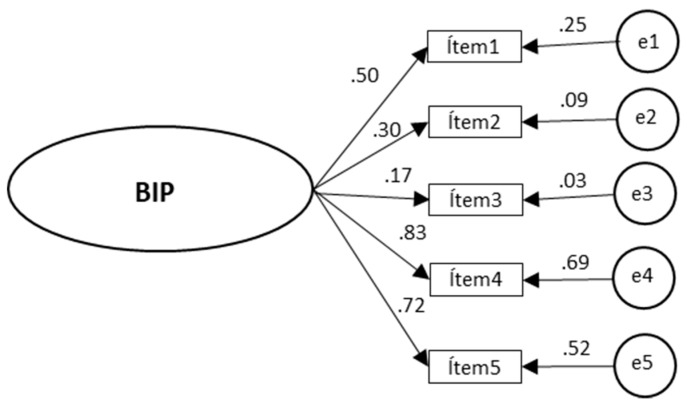
Confirmatory factor analysis of the BIP-Q5 model for COVID-19.

Although both the one-factor and two-factor versions of the original model showed adequate values, they could be improved. The one-factor model of the BIP-Q5, which consisted of a single factor and five items, was the most adequate once some respecifications had been analyzed considering theoretical and statistical criteria (change indices, errors of estimation, standard errors of measurement). The one-factor model of the BIP-Q5 then had a much better fit with the calibration sample. In addition, as the difference between the Akaike Information Criterion (AIC) default model = 40,167 and the AIC saturated model = 49,000 was very small, it is probably the best according to the Akaike criteria for model selection (Figure 2).

Finally, data from confirmatory factor analysis of the model proposed with the validation sample (*n* = 508) showed the following fit indices: *χ^2^/df =* 3.081, CFI = 0.973, TLI = 0.947, and RMSEA = 0.064 (0.029–0.102), which are adequate values. 

The reliability analysis of the model yielded a Spearman–Brown coefficient of *p* = 0.65 and Cronbach’s alpha with the whole sample was α = 0.66. The temporal stability analysis yielded an intraclass correlation coefficient (ICC) for perception of threat from COVID-19 of 65 and confidence interval = 0.62–0.69.

Table 4 shows the values for the six different models in the analysis of variance by sex, where it may be seen that in practically all cases, the ΔCFI was below 0.01, therefore configural, metric, and strict invariance may be accepted. Strong invariance may not be assumed, since the factor coefficients and intercepts for the two models evaluated were not equivalent. 

Table 5 includes the scales evaluating the level of threat in the Spanish population, and also by sex.

## 4. Discussion

This study was performed to adapt the BIP-Q5 questionnaire to the disease caused by the SARS-CoV-2 (COVID-19), and to acquire more information on the instrument’s factor structure and test reliability and validity for this disease in a sample of Spanish adults. 

The analyses performed revealed that the fit of the one-factor model of the BIP-Q5 was better than the two-factor model, showing the validity of the unidimensional model for evaluating the perception of threat from COVID-19. This result coincides with previous studies that have tested the validity and reliability of the BIP-Q5 in an adolescent Spanish population [24], and also with a larger number of studies done with adult populations in other countries using the brief version of the IPQ (BIP-Q) [22]. However, although correlations between residuals in the model enabled us to identify opportunities for improving the instrument, (mainly Item 3 since it was below 0.60 on most of the items on the questionnaire i.e., Items 1, 2, 4, and 5), the test–retest values were optimum, explaining the total for the scale of 43.87 of the variance in perception of the disease. Compared to the study by Valero-Moreno et al. [24], internal consistency of the Spanish version adapted to COVID-19 found with the Cronbach’s alpha coefficient was acceptable, but somewhat lower.

Some limitations of this study should be mentioned. Although this brief version of the IPQ can quickly evaluate perception of disease, in our case adapted to COVID-19, and it is useful in studies operating with large samples, it should be proven and validated in other cultures, where its validity has already been demonstrated for other diseases. Furthermore, during data acquisition, although self-report scales are commonly used in research, there may have been some associated social desirability biases. Another of the limitations is the sex distribution of the sample, which could be due to the sampling procedure, but keeping in mind the general characteristic of the Spanish population in this respect, it may be considered representative as there are also more women than men in the population. The research design was a cross-sectional study which did not allow some factors that may have affected participant response to be controlled for, such as access to communication media, local number of cases in the city where the participant resides, time when surveyed, past experience with pandemics, level of preparation, available social and family support, cultural context, and religious beliefs. Future studies could add information on the relationship of these variables to perception of COVID-19.

## 5. Conclusions

This study intended to examine the representation of COVID-19 disease in the Spanish culture using a version of the BIP-Q5 adapted to its perception. The exploratory and confirmatory factor analyses supported a one-factor model with five items which, in the set of analyses performed, was the one that showed the best psychometric properties. Internal consistency for the overall scale was acceptable. This brief version of the IPQ supported the factor structure of the test for measuring perception of threat of COVID-19, produced by the SARS-CoV-2 virus in an adult Spanish population. These findings are pioneer for this disease and can orient preventive intervention that enables psychological wellbeing and quality of life to be improved in situations similar to the pandemic.

## Figures and Tables

**Table 1:** Descriptive statistics. Calibration sample (*n* = 505).

Items	*n*	M	SD	Skewness		Kurtosis	
				Statistic	Std. Error	Statistic	Std. Error
BIP-Q5 1	505	7.66	2.11	−1.27	0.11	1.75	0.22
BIP-Q5 2	505	6.95	1.68	−0.65	0.11	0.61	0.22
BIP-Q5 3	505	2.02	1.86	2.1	0.11	3.8	0.22
BIP-Q5 4	505	7.75	2.17	−1.02	0.11	0.48	0.22
BIP-Q5 5	505	6.61	2.39	−0.51	0.11	−0.52	0.22

Note: BIP-Q5 = Brief Illness Perception Questionnaire; SD: standard deviation.

**Table 2:** Factor structure, communalities (h^2^) eigenvalues, Cronbach’s alpha and percentage of explained variance (*n* = 505). Extraction method: principal components factoring.

	F1	*h*2
Item 1	0.618	0.383
Item 2	0.607	0.369
Item 3	0.314	0.099
Item 4	0.817	0.667
Item 5	0.822	0.676
Percentage explained variance	43.87	
Kaiser–Meyer–Olkin	0.71	
Barlett’s sphericity	χ2(10) = 426.320, *p* < 0.000	
Cronbach’s alpha	0.663	

**Table 3:** Fit indices for the models proposed (calibration sample *n* = 505).

Model			CFI	TLI	RMSEA		
	χ2 (df)	χ2/df			RMSEA	CI 90%	
						Lower.	Upper
One-factor model of the BIP-Q5	10.2 (5)	2.1	0.988	0.975	0.045	0.000	0.085
Two-factor model of the BIP-Q5	9.999 (4)	2.49	0.986	0.964	0.055	0.010	0.098

Note: CFI = comparative fit index; TLI = Tucker–Lewis index; RMSEA = root mean square error of approximation; CI = confidence interval; *df* = degrees of freedom.

**Table 4:** Multigroup invariance by sex (male/female).

Model	χ2	df	χ2 / df	Δχ2	CFI	ΔCFI	IFI	RMSEA (CI 90%)
M0a (male)	29.129 (*p* = 0.001)	10	2.912		0.976		0.976	0.044 (0.026–0.063)
M0b (female)	29.129 (*p* = 0.001)	10	2.912		0.976		0.976	0.044 (0.026–0.063)
M1 (base model set)	29.129 (*p* = 0.001)	10	2.912		0.976		0.976	0.044 (0.026–0.063)
M2 (FS)	34.822 (*p* = 0.002)	14	2.487	0.424	0.973	0.003	0.974	0.039 (0.023–0.055)
M3 (FS + Int)	65.620 (*p* = 0.000)	19	3.453	0.966	0.940	0.033	0.940	0.050 (0.037–0.063)
M4 (FS + Int + Err)	76.002 (*p* = 0.000)	25	3.041	0.412	0.935	0.005	0.934	0.045 (0.034–0.057)

Note: FS = factor saturations, Int = intercepts, Err = errors.

**Table 5:** Scales for general population and by sex.

		General	Male	Female
	M	30.74	29.22	31.48
	*S*D	6.63	7.03	6.29
	Min	8	8	11
	Max.	50	50	48
**Percentiles**	10	21	20	23
	20	25	23	26
	30	28	27	29
	40	30	28	30
	50	31.5	30	32
	60	33	32	34
	70	34	33	35
	80	36	35	37
	90	38	37	39
	95	40	39	41
	99	45	43.66	45

## References

[1] Lai C.C., Shih T.P., Ko W.C., Tang H.J., Hsueh P.R. (2020). Severe acute respiratory syndrome coronavirus 2 (SARS-CoV-2) and coronavirus disease-2019 (COVID-19): The epidemic and the challenges. Int. J. Antimicrob. Agents.

[2] Lake M.A. (2020). What we know so far: COVID19 current clinical knowledge and research. Clin. Med..

[3] Wang F.S., Zhang C. (2020). What to do next to control the 2019-nCoV epidemic?. Lancet.

[4] Decaro N., Lorusso A. (2020). Novel human coronavirus (SARS-CoV-2): A lesson from animal coronaviruses. Vet. Microbiol..

[5] European Centre for Disease Prevention and Control An Agency of the European Union Situation Update Worldwide, as of 19 April 2020. https://www.ecdc.europa.eu/en/geographical-distribution-2019-ncov-cases.

[6] World Health Organization Coronavirus Disease (COVID-19) Pandemic. https://www.who.int/emergencies/diseases/novel-coronavirus-2019.

[7] Rabi F.A., Al Zoubi M.S., Kasasbeh G.A., Salameh D.M., Al-Nasser A.D. (2020). SARS-CoV-2 and Coronavirus Disease 2019: What we know so far. Pathogens.

[8] Rodríguez-Morales A.J., MacGregor K., Kanagarajh S., Patel D., Schlagenhauf P. (2020). Going Global- Travel and the 2019 Novel Coronavirus. Trop. Med. Infect. Dis..

[9] Ho C.S., Chee C.Y., Ho R.C. (2020). Mental Health strategies to combat the psychological impact of COVID-19 beyond paranoia and panic. Ann. Acad. Med. Singap..

[10] Hernández B.C., Rugarcía Y.T. (2015). Attitudes toward the risk prevention in health professionals in cases of epidemiological alert. Med. Segur. Trab..

[11] Lai J., Ma S., Wang Y., Cai Z., Hu J., Wei N., Wu J., Du H., Chen T., Li R. (2020). Factors Associated With Mental Health Outcomes Among Health Care Workers Exposed to Coronavirus Disease 2019. JAMA Netw. Open.

[12] Broadbent E., Wilkes C., Koschwanez H., Weinman J., Norton S., Petrie K.J. (2015). A systematic review and meta-analysis of the Brief Illness Perception Questionnaire. Psychol. Health.

[13] Johnson J., Hall L.H., Berzins K., Baker J., Melling K., Thompson C. (2018). Mental healthcare staff well-being and burnout: A narrative review of trends, causes, implications, and recommendations for future interventions. Int. J. Ment. Health Nurs..

[14] Molero M.M., Pérez-Fuentes M.C., Gázquez J.J., Simón M.M., y Martos Á. (2018). Burnout risk and protection factors in certified nursing aides. Int. J. Environ. Res. Public Health.

[15] Beléndez M., Bermejo R.M., García-Ayala M.D. (2005). Estructura factorial de la versión española del Revised Illness Perception Questionnaire en una muestra de hipertensos. Psicothema.

[16] Broadbent E., Petrie K.J., Main J., Weinman J. (2006). The brief illness perception questionnaire. J. Psychosom. Res..

[17] Moss-Morris R., Weinman J., Petrie K., Home R., Cameron L., Buick D. (2002). The revised illness perception questionnaire (IPQ-R). Psychol. Health.

[18] Taylor S. (2007). Psicología de la Salud.

[19] Lloyd K.R., Jacob K.S., Patel V., Louis L.S., Bhugra D., Mann A.H. (1998). The development of the Short Explanatory Model Interview (SEMI) and its use among primary-care attenders with common mental disorders. Psychol. Med..

[20] Rüdell K., Bhui K., Priebe S. (2009). Concept, development and application of a new mixed method assessment of cultural variations in illness perceptions barts explanatory model inventory. J. Health Psychol..

[21] Quiceno J.M., Vinaccia S. (2010). Percepción de enfermedad: Una aproximación a partir del Illness Perception Questionnaire [Illness perception: An approximation from the Illness Perception Questionnaire]. Psicol. Caribe.

[22] Karataş T., Özen Ş., Kutlutürkan S. (2017). Factor structure and psychometric properties of the brief illness perception questionnaire in Turkish cancer patients. Asia Pac. J. Oncol. Nurs..

[23] Zhang N., Fielding R., Soong I., Chan K.K., Lee C., Ng A., Wing K., Janice T., Lee V., Wendy W. (2017). Psychometric assessment of the Chinese version of the brief illness perception questionnaire in breast cancer survivors. PLoS ONE.

[24] Valero-Moreno S., Lacomba-Trejo L., Casaña-Granell S., Prado-Gascó V.J., Montoya-Castilla I., Pérez-Marín M. (2020). Propiedades psicométricas del cuestionario de percepción de amenaza de la enfermedad crónica en pediatría. Rev. Lat. Am. Enferm..

[25] Leventhal H., Meyer D., Nerenz D.R., Rachman S. (1980). The common sense representation of illness danger. Contributions to Medical Psychology.

[26] Leventhal H., Phillips L.A., Burns E. (2016). The Common-Sense Model of Self-Regulation (CSM): A dynamic framework for understanding illness self-management. Int. J. Behav. Med..

[27] Pacheco-Huergo V., Viladrich C., Pujol-Ribera E., Cabezas-Pena C., Núnez M., Roura-Olmeda P., Amado-Guiradoh E., Núñez E., del Val J.L. (2012). Percepción en enfermedades crónicas: Validación lingüística del Illness Perception Questionnaire Revised y del Brief Illness Perception Questionnaire para la población española. Aten. Prim..

[28] Chew B.H., Vos R.C., Heijmans M., Shariff-Ghazali S., Fernandez A., Rutten G.E. (2017). Validity and reliability of a Malay version of the brief illness perception for patients with type 2 diabetes mellitus. BMC Med. Res. Methodol..

[29] Leysen M., Nijs J., Meeus M., Van Wilgen C.P., Struyf F., Vermandel A., Kuppens A., Roussel N. (2015). Clinimetric properties of illness perception questionnaire revised (IPQ-R) and brief illness perception questionnaire (Brief IPQ) in patients with musculoskeletal disorders: A systematic review. Man. Ther..

[30] Quiles Y., Terol M.C., Tirado S., Beléndez M. (2007). Estructura factorial de la versión española del “Cuestionario de Percepción de Enfermedad Revisado” (IPQ-R) en pacientes con un trastorno del comportamiento alimentario y sus familiares. Cuad. Med. Psicosom. Psiquiatr. Enlace.

[31] Pérez-Fuentes M.C., Herrera-Peco I., Molero M.M., Oropesa N.F., Ayuso D., Gázquez J.J. (2019). The Development and Validation of the Healthcare Professional Humanization Scale (HUMAS) for Nursing. Int. J. Environ. Res. Public Health.

[32] Bentler P.M. (1989). EQS Structural Equations Program Manual.

[33] McDonald R.P., Ho M.H.R. (2002). Principles and practice in reporting structural equation analyses. Psychol. Methods.

[34] Akaike H. (1974). A new look at the statistical model identification. IEEE Trans. Automat. Control.

[35] Cronbach L.J. (1951). Coefficient alpha and the internal structure of tests. Psychometrika.

[36] Byrne B.M., Stewart S.M. (2006). The MACS approach to testing for multigroup invariance of a second-order structure: A walk through the process. Struct. Equ. Model..

[37] Cheung G.W., Rensvold R.B. (2002). Evaluating goodness-of-fit indexes for testing measurement invariance. Struct. Equ. Model..

[38] Finney S.J., di Stefano C., Hancock G.R., Mueller R.O. (2006). Non-normal and categorical data in structural equation modeling. Structural Equation Modeling: A Second Course.

